# Chicago College of Pharmacy

**Published:** 1867-03

**Authors:** 


*Chicago College of Pharmacy.*—We chronicle with great pleasure the action of the leading druggists of this City in reviving this organization. At a recent meeting, largely attended, and at which a commendably enthusiastic spirit was manifested, the following officers were elected for the ensuing year:—

*President*—Mr. E. H. SARGENT.

*Vice-Presidents*—Messrs GEORGE BUCK and W. H. MULLER.

*Secretary*—Mr. JAMES W. MILL.

*Treasurer*—Mr. J. P. SHARP.

*Trustees*—ALBERT E. EBERT, HENRY SWEET, JOHN W. EHRMAN, WILLIAM REINBOLD, and EMIL DREIER.

A costly and extensive collection of chemical specimens were present for exhibition, having been presented the College by Messrs. POWERS & WEIGHTMAN, the well-known manufacturing chemists of Philadelphia.

Permanent rooms have been secured for the use of the Association, which are open for the observation of the Medical profession, as well as the druggists of the country. We cordially endorse the language of our reporter:—

"The large attendance, the enthusiastic and hopeful spirit manifested at this meeting, augur well for the future success of the College. The other large cities of the Union—Philadelphia, New York, Boston, Baltimore, Cincinnati, and St. Louis—have their Colleges of Pharmacy. Why not Chicago? Let the druggists of Chicago have regard to their reputation for intelligence and enterprise, and see to it that the impetus thus given to their College does not fail, from any supineness or indifference on their part, to carry it forward to a high position of usefulness and honor. It is to be hoped that not only the druggists of the City, but many, also, throughout the Northwest, will feel sufficient interest to identify themselves with the

College, either as active or associate members. Copies of the constitution and by-laws can be had on application to the Secretary, JAMES W. MILL, 119 West Adams Street."

